# A Common 3′UTR Variant of the *PHOX2B* Gene Is Associated With Infant Life-Threatening and Sudden Death Events in the Italian Population

**DOI:** 10.3389/fneur.2021.642735

**Published:** 2021-03-19

**Authors:** Tiziana Bachetti, Simona Bagnasco, Raffaele Piumelli, Antonella Palmieri, Isabella Ceccherini

**Affiliations:** ^1^Laboratorio di Neurobiologia dello Sviluppo, Dipartimento di Scienze della Terra, dell'Ambiente e della Vita (DISTAV), Università di Genova, Genoa, Italy; ^2^Laboratorio di Genetica e Genomica delle Malattie Rare, Istituto di Ricerca e Cura a Carattere Scientifico (IRCCS) Giannina Gaslini, Genoa, Italy; ^3^Centro per i Disturbi Respiratori nel Sonno-Centro Regionale SIDS, Ospedale Meyer, Florence, Italy; ^4^Dipartimento di Emergenza, Centro SIDS-ALTE, Istituto di Ricerca e Cura a Carattere Scientifico (IRCCS) Giannina Gaslini, Genoa, Italy

**Keywords:** *PHOX2B*, sudden infant death syndrome, idiopathic apparent life threatening event, miR-204, gene expression regulation, sudden unexpected infant death

## Abstract

Heterozygous mutations in the Paired like homeobox 2b (*PHOX2B*) gene are causative of congenital central hypoventilation syndrome (CCHS), a rare monogenic disorder belonging to the family of neurocristopathies and due to a defective development of the autonomic nervous system. Most patients manifest sudden symptoms within 1 year of birth, mainly represented by central apnea and cyanosis episodes. The sudden appearance of hypoxic manifestations in CCHS and their occurrence during sleep resemble two other unexplained perinatal disorders, apparent life-threatening event (ALTE) and sudden and unexpected infant death (SUID), among which the vast majority is represented by sudden infant death syndrome (SIDS). Differently from CCHS, characterized by Mendelian autosomal dominant inheritance, ALTE and SIDS are complex traits, where common genetic variants, together with external factors, may exert an additive effect with symptoms likely manifesting only over a “threshold.” Given the similarities observed among the three abovementioned perinatal disorders, in this work, we have analyzed the frequency of *PHOX2B* common variants in two groups of Italian idiopathic ALTE (IALTE) and SUIDs/SIDS patients. Here, we report that the c^*^161G>A (rs114290493) SNP of the 3′UTR *PHOX2B* (i) became overrepresented in the two sets of patients compared to population matched healthy controls, and (ii) associated with decreased *PHOX2B* gene expression, likely mediated by miR-204, a microRNA already known to bind the 3′UTR of the *PHOX2B* gene. Overall, these results suggest that, at least in the Italian population, the SNP c^*^161G>A (rs114290493) does contribute, presumably in association with others mutations or polymorphisms, to confer susceptibility to sudden unexplained perinatal life-threatening or fatal disorders by increasing the effect of miR-204 in inducing *PHOX2B* expression down-regulation. However, these are preliminary observations that need to be confirmed on larger cohorts to achieve a clinical relevance.

## Introduction

Paired like homeobox 2b (*PHOX2B*) gene acts in the early development of the autonomic nervous system (ANS), regulating the expression of downstream genes that lead to neuronal differentiation (1). *PHOX2B*, expressed in several districts of the ANS, controls the response to CO_2_ by integrating chemosensory information derived from carotid body, the nucleus of Tractus Solitarius (NTS), and the Retro Trapezoid Nucleus (RTN) (2, 3). Heterozygous mutations of the *PHOX2B* gene are responsible for congenital central hypoventilation syndrome (CCHS), a rare autosomal dominant genetic condition characterized by impaired response to hypercapnia and hypoxia due to compromised autonomic control of breathing (4–6). In particular, causative mutations are represented by triplet tandem duplications in exon 3 leading to polyalanine (polyAla) expansions, accounting for 90% of CCHS cases, in addition to missense, non-sense, and frameshift mutations responsible for the remaining patients.

CCHS can occur either isolated or in association with other neural crest-derived disorders such as tumors of the sympathetic nervous system (TSNS) and Hirschsprung disease (HSCR), in which not only causative mutations but also *PHOX2B* common variants have been shown to play a role as susceptibility genetic factors (7, 8). CCHS, with the exceptions of rare late onset forms, manifests at birth with central apnea leading to cyanosis, hypercapnia, and desaturation during sleep and can be considered a “chronic” condition persisting lifetime (9). Most patients need to be managed by ventilation only during asleep; however, in a small percentage of cases carrying the largest polyAla expansions, ventilation support is also needed while awake (9).

Interestingly, at symptom onset, CCHS may be viewed as an apparent life-threatening event (ALTE). ALTE is defined as an episode that scares the observer and is characterized by one or more of the following symptoms: (i) apnea (central or occasionally obstructive); (ii) color change (usually cyanotic or pallid but occasionally erythematous or plethoric); (iii) marked change in muscle tone (usually marked limpness); (iv) altered level of responsiveness. This term indicates high-risk conditions leading to the need of resuscitation actions performed by a caregiver, while the more recent term “brief resolved unexplained event” (BRUE) indicates short-lived events that resolve spontaneously and allow patients to be discharged home after a few hours (10). Clinical and/or molecular analyses can disclose a specific etiology in 50–70% ALTE cases, the remaining unresolved events being defined idiopathic ALTE (IALTE) (11).

Previously, these episodes were referred to as “near-miss” sudden infant death syndrome (SIDS) (12). SIDS represents 80% sudden and unexpected infant death (SUID), a fatal event characterized by unexplained death, usually during sleep, of a seemingly healthy baby less than a year old. The incidence of SIDS increases 5-fold in families that had a previous SIDS case (13), thus suggesting that a genetic basis underlies this condition. However, differently from the monogenic CCHS, and according to the so called “triple risk hypothesis,” SIDS is regarded to be a complex disorder, in which predisposing genetic factors likely act in concert with external circumstances in a critical developmental period (14). Interestingly, a common variant in the Serotonin Transporter 5-HTT promoter was similarly associated with both SIDS and IALTE (15), thus suggesting a common genetic predisposition in sudden infant death and not clinically explained life-threatening events.

In this light, the early manifestations of CCHS can be seen in ALTE events except that a genetic diagnosis can be achieved later in the former but not in the latter conditions. This has suggested a role of the *PHOX2B* gene in CCHS companion diseases such as SIDS and ALTE. Indeed, a whole PHOX2B gene deletion has been detected in a patient with ALTE (16) and, in different populations, *PHOX2B* common variants have been identified in association with SIDS (17, 18). In addition, brainstem developmental alterations have been identified both in CCHS (19) and in SIDS (20), where *PHOX2B* was shown less expressed and also found in the cytoplasm rather than in the nuclear compartment (21). All these evidences suggest that the etiology of SIDS/SUID, ALTE, and CCHS could share some genetic features among which *PHOX2B* plays a crucial role.

In this manuscript, we are reporting for the first time a genetic screening of the three exons of the *PHOX2B* gene in Italian IALTE and SIDS/SUID cases. A statistically significant association of common *PHOX2B* variants, namely, the rs17885216 (c.552C>T) SNP in exon 3, found in linkage disequilibrium with the rs114290493 (c.^*^161G>A) SNP in the *PHOX2B* 3′UTR, is reported for the conditions mentioned above. Moreover, *in silico* and functional analyses of possible effects of these two variants were performed.

## Methods

### Patients Collection and DNA Extraction

Patients have been collected by the SIDS-ALTE Center of Liguria Region (Italy) and the SIDS Center of Meyer Institute (Italy) from 2010 to 2020 (22). Among them, only Italian cases were considered for this work. Informed consent was obtained from patients' parents, and patients' DNA was extracted from either peripheral blood or available autoptic specimens from SUIDs/SIDS, following standard laboratory procedures.

Only individuals lacking any clinical diagnosis, made after the sudden episodes, have been included in the analysis. In particular, 12 IALTE patients and 7 unexpected dead infants, including 6 SUID and 1 SIDS, were considered. Seventy-one DNA control samples, obtained from healthy donors of the Istituto Gaslini and matched for sex but not for age, were also analyzed.

### *PHOX2B* Gene Analysis

The mutation screening of the *PHOX2B* gene (GenBank NM_003924.3) was performed as already reported (6). In particular, the three *PHOX2B* exons were amplified by specific primers (Supplementary Table 1) by using the GC Rich PCR System (Roche). Reaction mixes were run for 35 cycles at 95°C denaturation for 1 min, 60°C annealing for 45 s, and 72°C extension for 1 min and 30 s.

PCR fragments were purified with the SapI–ExoIII enzymatic mix by incubating at 37°C for 40′ and at 80°C for 15′ and analyzed for mutations by direct DNA sequencing using the Big Dye Terminator Cycle Sequencing Kit (Applied Biosystem) on an ABI 3100 DNA automated sequencer.

The *PHOX2B* 7Ala in-frame deletion (hereon 7Ala contraction) was confirmed also by using the “FAM method” (23). In detail, PCR was performed with 22F-FAM 5′-CTGACCCGGACAGCACTGGGGGCC-3′, 5′ end-labeled with FAM, and 279R 5′-GAGCCCAGCCTTGTCCAGG-3′ by the Accuprime GC kit (Life Technologies). Reaction mixes were run for 35 cycles at: 95°C denaturation for 1 min, 62°C annealing for 45 s, and 72°C extension for 45 s, followed by 20 min final extension. One microliter of the PCR product was mixed to 12 μl of formamide and 0.3 μl of ROX 500 size marker (Applied Biosystems) and loaded on the ABI 3100 DNA automated sequencer. Data were then analyzed by GeneMapper (Applied Biosystems).

### Evaluation of the SNPs Allele Phase

DNA samples carrying both SNPs rs17885216 (c.552C>T) in exon 3 and rs114290493 (c.^*^161G>A) in 3′UTR were amplified with primers: 10F: 5′-TGCTTCACCGTCTCTCCTTCC-3′ and PH2B-3UTR (2) 5′-ATCAGCAGGCGGAGCCC-3′. The product thus obtained, encompassing both loci, was cloned into pCR2.1 (TOPO TA cloning kit, Life Technologies). Colonies were grown in LB/Ampicillin medium, and the plasmids thus obtained, each containing one of the two allele combinations, were sequenced with the primers above to assess the phase of the alleles.

### Statistical Analysis

Fisher's exact test was applied to a 2 × 2 contingency table to evaluate statistical significance of allele frequencies between groups under analysis. Allele frequencies of *PHOX2B* SNPs assessed from a panel of 71 Italian healthy donors were used for comparing cases and controls.

### *In silico* Tools for Predicting *PHOX2B* mRNA Post-transcriptional Modifications

*In silico* prediction of splicing was assessed by using both Alternative Splice Site Predictor (ASSP) (http://wangcomputing.com/assp/) (24) and ESE-Finder 3.0 (http://krainer01.cshl.edu/cgi-bin/tools/ESE3/esefinder.cgi) (25).

For microRNA prediction, MicroRNA Target Prediction Database (miRDB) (http://mirdb.org/custom.html) was used by applying the custom prediction option, while TargetScan 5.2 (http://www.targetscan.org) was used to evaluate conserved sites.

### Construction of c^*^161G and c^*^161A (rs114290493) Reporter Plasmids and IMR32 Transfections

A 219-bp region of the 3′UTR *PHOX2B* encompassing the c^*^161G>A (rs114290493) locus was amplified from a heterozygous SIDS patient. The PCR product was cloned into the pCR2.1 vector (TOPO TA cloning, Life Technologies), sequenced, and, after SacI–XhoI digestion, transferred downstream the firefly *luciferase* gene of the reporter pmirGlo Dual-Luciferase miRNA Target Expression vector (Promega), containing both firefly *luciferase* gene, whose expression is regulated by the exogenous subcloned region, and renilla *luciferase* gene, constitutively expressed and used as internal control.

To evaluate the effect of the rs11429049 c^*^161 G and A alleles, 150,000 human neuroblastoma IMR32 cells were transfected by Lipofectamine 2000 with 500 ng of pmirGlo plasmids and added at the time of transfection with 50 nM miR-204 mimic or negative control mir(C–) (Dharmacon). After 48 h, Luciferase detection was performed by the Dual Luciferase Reporter System (Promega) with a TD20/20 luminometer (Turner Designs).

## Results

### PHOX2B Screening in IALTE and SIDS/SUID

**Figure 1:**
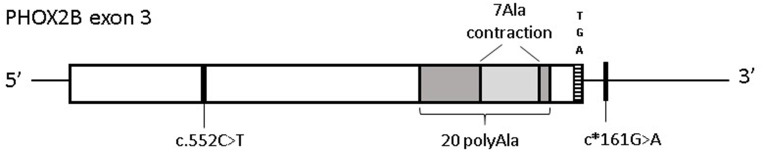
Distribution of PHOX2B exon 3 variants in Italian IALTE and SIDS/SUID patients. Graphical representation of the common variants in *PHOX2B* exon 3 identified in ALTE and SIDS/SUID cases. Black line indicates the position of the synonymous variant c.552C>T (p.184Ser=) and c*161G>A, in linkage disequilibrium with c.552C>T; the light gray box represents the 7 polyAla contraction within the 20 polyAla region, shown in dark gray. The TGA stop codon indicates the end of the coding region.

To search for *PHOX2B* variants that could underlie severe perinatal conditions with, similarly to CCHS, alterations of the autonomous nervous system, the three *PHOX2B* exons have been analyzed in 12 IALTE (7 females and 5 males) patients and a group of 7 unexpected dead infants, including 6 SUID and 1 SIDS (2 females and 5 males). Although no causative variants were identified, we found common variants including a single-nucleotide polymorphism leading to synonymous changes c.552C>T; p.184Ser=(rs17885216) and an in-frame deletion of 7 alanine residues (hereon 7 Ala contraction), shortening the 20 polyalanine (PolyAla) stretch to final 13 Ala residues, in *PHOX2B* exon 3 (Figure 1).

In particular, among the four c.552C>T alleles, two were found in IALTE and two were found in the SUID/SIDS group, the 7 Ala contraction was found in an IALTE sample.

To assess a possible difference between minor allele frequencies (MAF) observed in our cases compared to the healthy population, we performed a Fisher's exact test. In particular, an “in-house” panel of 71 DNAs from Italian healthy donors, recruited in the Giannina Gaslini Institute, was used for frequency allele comparison. While the 7 Ala contraction did not differ significantly from the expected frequency, SNP c.552C>T variant allele was statistically more frequent in both IALTE and SIDS/SUID groups compared to controls. The association became even more significant when these two groups were merged (Table 1). Of note, by comparing European-non-Finnish allelic frequencies retrieved from the GnomAD v.2.1.1 browser (https://gnomad.broadinstitute.org/), we obtained statistically significant *p* values for each group (IALTE = 0.029; SUIDs/SIDS = 0.01; IALTE+SUIDs/SIDS = 0.0008).

**Table 1:** Minor allele frequency of the *PHOX2B* common variants found in IALTE and SUIDs/SIDSs cases.

		**IALTE (*****n*** **= 12)**			**SUIDs/SIDS (*****n*** **= 7)**			**IALTE+SUIDs/SIDS (*****n*** **= 19)**			**Ctrls (*****n*** **= 71)**	
		**Minor allele**			**Minor allele**			**Minor allele**			**Minor allele**	
**ID**	**Variants^**§**^**	***N***	***F***	***p*-value**	***N***	***F***	***p*-value**	***N***	***F***	***p*-value**	***N***	***F***
rs17885216rs114290493	c.552C>T c.^*^161G>A	2	0.08	0.05^*^	2	0.14	0.02^*^	4	0.1	0.007^*^	1	0.014
rs746486633	7 Ala contraction	1	0.04	0.36	0	0	na	1	0.03	0.51	2	0.01
TOT/cumulative frequency	3	0.125	0.06	2	0.14	0.15	5	0.13	0.02^*^	4	0.03

§ Variant alleles of SNPs c.552C>T and c.

* 161G>A are in linkage disequilibrium; N, number of minor alleles; F, allele frequency; p-value, asterisks (*) indicate statistically significant values with respect to control samples; na, not assessable.

The c.552C>T SNP was reported in the Ensemble Variant database (http://www.ensembl.org/info/genome/variation/index.html) in complete linkage disequilibrium with a variant in the *PHOX2B* 3′UTR, the c.^*^161G>A (rs114290493) SNP. Analysis of this latter variant showed that all IALTE and SIDS cases carrying the c.552C>T SNP were heterozygous also for the variant c.^*^161G>A (26), the two variants sharing in fact the same allelic frequency (0.008) (https://gnomad.broadinstitute.org/region/4-41746099-41750987?dataset=gnomad_r2_1) (27) (Figure 1 and Table 1). The *cis* phase of these two variant alleles was confirmed by subcloning a fragment containing both loci and showing that the two haplotypes included either the reference alleles c.552C-c.^*^161G (CG) or the variant alleles c.552T-c.^*^161A (TA). Given the presence of the TA haplotype in IALTE and SIDS DNA samples, we wondered whether its effect could be mediated by gene expression regulation mechanisms able to modify the amount of *PHOX2B* allele product. To this end, we have investigated possible post-transcriptional modifications (PTMs) by using *in silico* tools and performing suitable functional tests.

### *In silico* Prediction of the Effects of the c.552C>T and c.^*^161G>A Variants

As splicing alterations have emerged as a mechanism affecting gene expression regulation (28), we wondered whether the T variant allele of the c.552C>T SNP could alter the exon–exon junctions. Two *in silico* tools, ESE-finder 3.0 and Alternative Splice Site Predictor (ASSP), predicted the T allele to interfere at some extent with the splicing by altering both the 3′ splice site (3SS) and the branch site (Supplementary Tables 2, 3). However, as the c.552C>T SNP is in the third and last *PHOX2B* exon, the “exon trapping” experimental approach usually carried out using the minigene vector pSPL3 (29) turned out to be ineffective to study the splicing, because of the lack of the GT donor site downstream the exon under analysis (data not shown).

**Figure 2:**
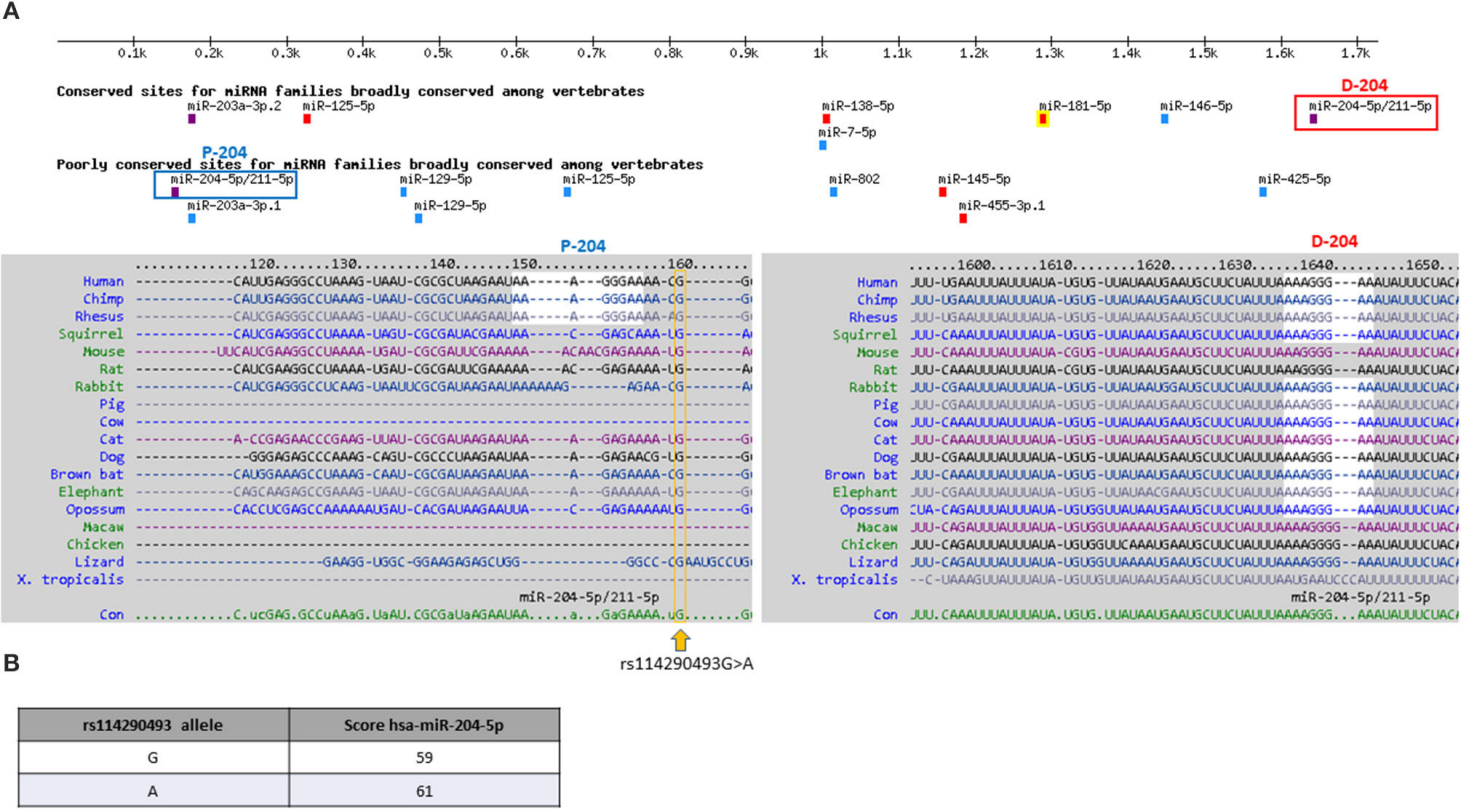
*In silico* prediction of microRNAs binding 3′UTR PHOX2B. **(A)** Target scan release showing microRNA binding the 3′UTR of the *PHOX2B* gene; in the upper part of the figure, the color scale indicates the strength of the prediction, based on the specificity of the seed (violet: 8mer; red: 7mer-m8; cyan: 7mer-A1; green: non-canonical). In the lower part of the figure, the sequences surrounding the proximal, less conserved miR204 site (P-204) and the distal, more conserved miR204 site (D-204) are shown. The c.*161G>A position is boxed in yellow. **(B)** The table shows results obtained by the custom prediction option MicroRNA Target Prediction Database (miRDB); results show the different miR-204 affinity in the presence of the c.*161 G and A alleles. In the two columns, the allele and the score of miR-204 binding are shown.

We then verified whether *PHOX2B* gene expression could be modified by the common variant c.^*^161A in the 3′UTR *PHOX2B*, in linkage disequilibrium with the above c.552T variant. In the 3′UTR *PHOX2B*, two sequences are predicted to be bound by miR-204 (Figure 2A), one “distal” (D-204), lying within a highly conserved region of the 3′UTR and classified as high conserved miR-204 binding site, the other one “proximal” (P-204), lying within a moderately conserved region of the 3′UTR and classified as low conserved miR-204 binding site (Figure 2A). The miR-204-mediated *PHOX2B* expression regulation has already been demonstrated through the D-204 site, together with the effect of the SNP rs1063611 flanking the same D-204 site (26) (Supplementary Figure 1). As the SNP c^*^161G>A is very close to the P-204 site, we checked whether it could display a similar effect. Preliminary *in silico* analysis performed to search for microRNA differently regulating the two alleles showed that, in the presence of the variant c.^*^161A allele, miR-204 is predicted with a score higher than in presence of the c.^*^161G allele (Figure 2B), thus suggesting that the A allele could induce a reinforced miR-204 mediated down-regulation of the *PHOX2B* gene expression with respect to the G allele.

### *In vitro* Prediction of the c.^*^161G>A (rs114290493) SNP Consequences

**Figure 3:**
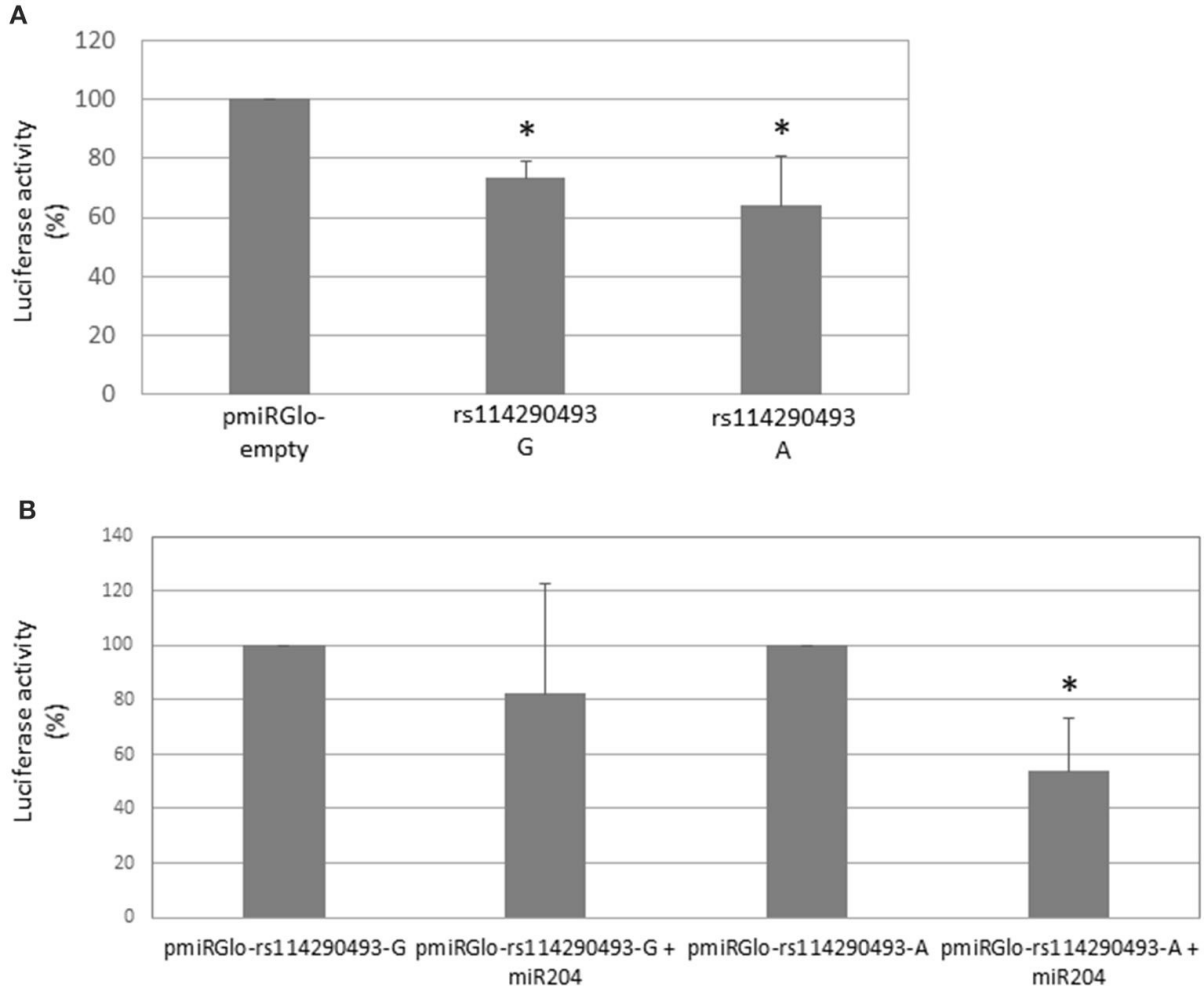
Effects of rs114290493 alleles on miR-204 mediated expression modulation. **(A)** The effect of the 219-bp subcloned region on the Luciferase stability is shown in the presence of both G and A alleles; values are expressed as percentage of the empty pmiRGlo vector expressed as 100 and are the mean of three independent experiments performed in triplicate ±SD. Asterisks (*) indicate statistically significant values (Student's *t*-test, *p* < 0.05) obtained by comparing each allele with respect to the empty vector (*p-value G: 0.015; *p*-value A: 0.019). **(B)** The bar graph shows the Luciferase activity induced by addition of miR-204 as percentage of the value obtained in the absence of miR-204, referred as 100. Values are the mean of three independent experiments performed in triplicate ±SD. Asterisk (*) indicates statistical significance (Student's *t*-test, *p* < 0.05) obtained by comparing each allele added vs. not added with miR-204 (*p value A: 0.0001).

In order to evaluate the effects of the PHOX2B c.^*^161G>A 3′UTR variant, a 219-bp region amplified from a SIDS patient heterozygous at this locus was cloned in the pmiRGLo reporter plasmid downstream the firefly *luciferase* gene and the construct thus obtained transfected in the IMR32 human neuroblastoma cell line. First, we could observe that this sequence, independently of the c.^*^161 allele present, induced a decrease in the Luciferase activity with respect to the empty vector, thus suggesting that the sequence cloned was able to drive the down-regulation of the *luciferase* gene expression, thus confirming the suitability of the *in vitro* system (Figure 3A). Moreover, the comparison of the miR-204 effect induced on both the G and A constructs showed that addition of miR-204 to the A allele significantly reduced the Luciferase's activity, while it was not able to exert a significant effect on the G allele (Figure 3B). To exclude an effect of miR-204 on plasmid backbone, we compared values obtained from the co-transfection of miR-204 with the c.^*^161 G or A plasmids with values obtained from the co-transfection of miR-204 with the pmiRGlo empty vector. As shown in Supplementary Figure 2, overexpression of miR-204 together with the c.^*^161 plasmids was able to induce a decrease of Luciferase's activity compared to the empty pmiRGlo vector added with the same miRNA, for both the G and A plasmids, the effect on this latter allele being significantly stronger than on the former allele. Both alleles are therefore bound by miR-204, the A allele being more efficient than the G allele. However, when we compared the effects of miR-204 with those of the co-transfection with the negative control miR(C–) on the same c.^*^161 allele, we observed that the G allele was not modulated by miR-204 while the A allele was confirmed to be down-regulated by miR-204 addition (Supplementary Figure 2B). Our results confirmed the hypothesis that the c.^*^161G>A SNP variant might play a role in *PHOX2B* gene expression regulation mediated by miR-204. Moreover, such an effect was not in contrast with results obtained without miR-204 given that IMR32 cells are characterized by very low miR-204 endogenous levels (30), likely not sufficient to disclose such an allele-specific effect.

## Discussion

In this manuscript we have reported a *PHOX2B* variant screening performed in Italian IALTE and SUID/SIDS patients to search for genetic elements predisposing to these autonomic disorders, whose sudden manifestation may resemble CCHS, caused by *PHOX2B* mutations. While *PHOX2B* has already been analyzed in SIDS (17, 18, 31, 32), it has never been investigated in IALTE so far. Overall, this work represents the first analysis of the *PHOX2B* gene in Italian SIDS and IALTE cases. Results thus obtained showed a statistically significant association between these two groups and the single-nucleotide polymorphism rs17885216 leading to the synonymous nucleotide change c.552C>T in the exon 3 (p.Ser184=). Worldwide, the frequency of the c.552T allele spans from zero (absent) in East Asian, through 0.006718 in Latins, to the highest 0.018 in Africans. For this reason, a matched control population should be used to calculate the statistical significance of the association. This is true also for other *PHOX2B* polymorphisms, such as the intronic IVS2+101A>G (rs28647582) SNP as well as polyAla contractions, which became associated or not associated with SIDS according to the different population ethnicity (17, 18, 32). Similarly, the cumulative frequencies of PHOX2B common variants turned out to be statistically significant in the Caucasian SIDS population but not in African-American patients (17). Interestingly, *PHOX2B* common variants resulted in a cumulative frequency higher in a set of children affected by obstructive sleep apnea (OSA) with class III malocclusion than in controls (33), an observation that further supports the role of the *PHOX2B* gene in respiratory disorders beyond CCHS, as suggested by OSA occurrence in infants of families with multiple histories of SIDS and ALTE (34). Moreover, the cumulative frequency of *PHOX2B* common variants could reach a statistical significance also in our present Italian IALTE and SUIDS/SIDS cases.

Despite the idea that c.552C>T SNP is predicted to be benign or likely benign by the Ensembl Variation database https://www.ensembl.org/Homo_sapiens/Gene/Variation_Gene/Table?db=core;g=ENSG00000109132;r=4:41744082-41748725, a pathogenic role for this variant was sought. *In silico* prediction did suggest that this variant might impair the *PHOX2B* post-transcriptional regulation through an effect on splicing that however could not be assessed using a standard minigene system.

In addition, to evaluate the c.552C>T change, we have searched also for additional variants that could account for the observed association between common *PHOX2B* variants and Italian SIDS and IALTE cases. The identification of the linkage disequilibrium between the c.552C>T and the c^*^161G>A SNPs in the *PHOX2B* 3′UTR suggested the following possible scenario: (i) both polymorphisms act in concert to reduce *PHOX2B* expression, (ii) only one of the two is the effective variant while the other is a tag-SNP, and (iii) both SNPs belong to a haplotype where a still unidentified functional variant lies. A functional analysis performed using a Luciferase reporter construct has demonstrated that the c.^*^161A allele is able to reduce, alone, the *PHOX2B* expression at a higher extent than the c.^*^161G allele, thus suggesting that this variant induces a loss-of-function effect leading to decreased allele-specific gene expression and therefore to haploinsufficiency. In particular, *in vitro* transfections of the two c.^*^161 allele reporter constructs did not reveal any effect of miR-204 on the G allele, while the presence of the A allele was always associated with a marked decrease in Luciferase's activity, likely reflecting its role on the entire 3′UTR *PHOX2B*. To perform such studies, human neuroblastoma IMR32 cells were used as expressing high *PHOX2B* levels and already demonstrated suitable to study *PHOX2B* post-transcriptional regulation (26). In particular, they are characterized by a relatively low miR-204 expression with respect to non-MYCN amplified neuroblastoma cell lines (30), an ideal condition to investigate the effects of this microRNA not only in tumors of the sympathetic nervous system but also in neurodevelopmental conditions. The miR-204 consensus on the PHOX2B 3′UTR was not disrupted by site-directed mutagenesis assays, we could not definitively confirm the miR-204 binding to the proximal site of PHOX2B 3′UTR. Nevertheless, the different effects of miR-204 on the two PHOX2B alleles confirm that, independently of whether directly or indirectly, miR-204 does act also on this proximal site.

**Figure 4:**
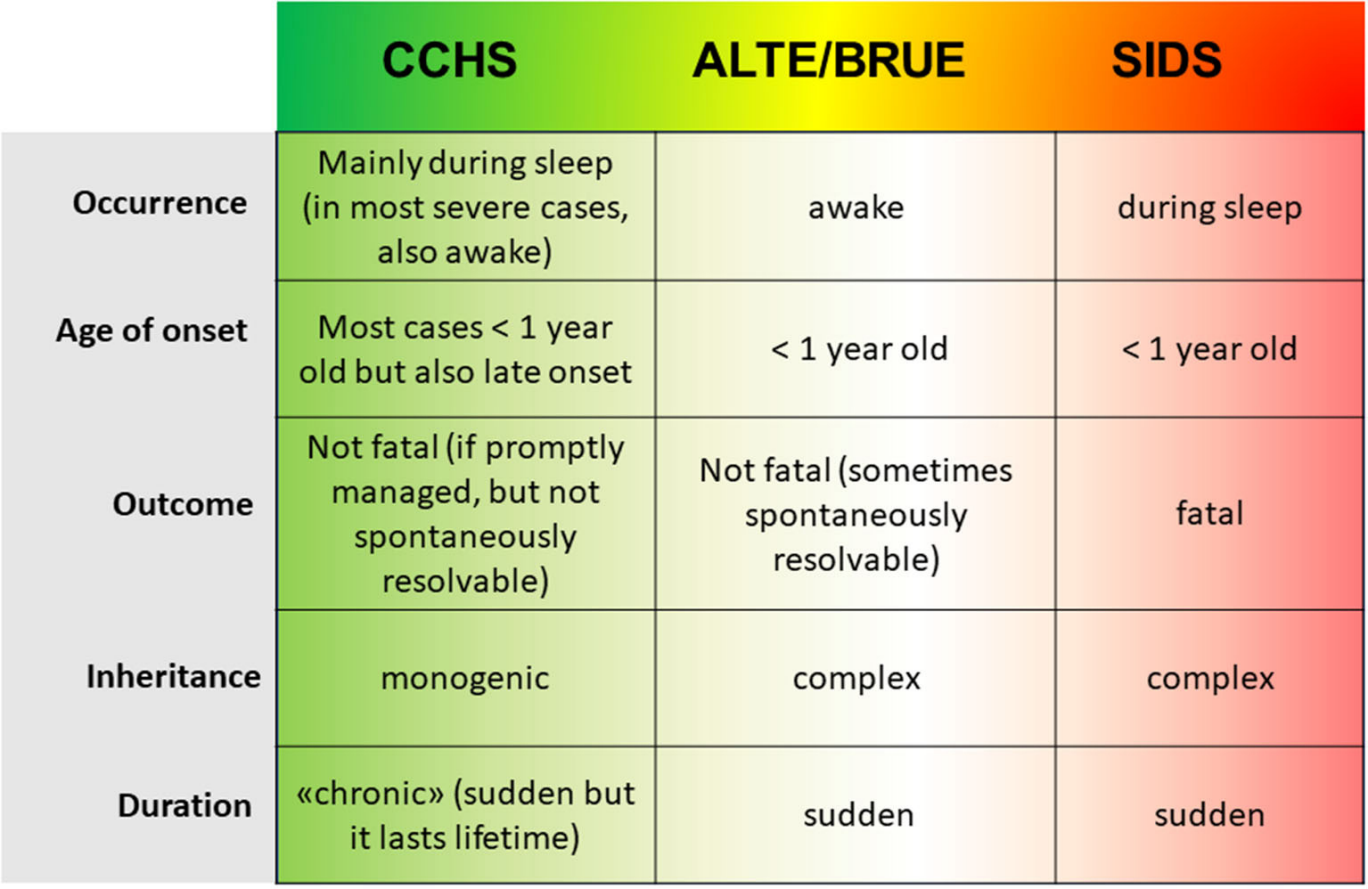
The scheme represent the clinical and genetic features of the three condition CCHS, ALTE, and SIDS; from left to right, starting from the less severe monogenic CCHS to the fatal complex SIDS, the increase in severity is represented by the color-scale green (CCHS)-yellow (ALTE/BRUE)-red (SIDS).

Taken together, our present results are in accordance with observations made in neurons from SIDS specimens, where *PHOX2B* expression has been found to be lower than expected (21), thus strengthening the hypothesis of a loss-of-function effect of *PHOX2B* variants in SIDS. The similarity between IALTE and SIDS relies also on the identification of the L/L genotype of the serotonin transporter (5-HTT) polymorphism in both these disorders (15), thus suggesting that they might be different manifestations of a common etiopathogenesis, with SIDS events resembling IALTE episodes occurred during sleep, out of parental control. Therefore, CCHS, ALTE, and SIDS might be members of the same group of respiratory and autonomic disorders of infancy and, along an imaginary severity line, starting from the “chronic” CCHS to the fatal SIDS, they might belong to a same sudden perinatal disorder spectrum (Figure 4), with *PHOX2B* variants playing a causative role in CCHS and predisposing to the two other disorders. Taken together, these results confirm that the haplotype including the c.552C>T (rs17885216) and c^*^161G>A (rs114290493) variant alleles is associated with IALTE and SUID/SIDS. Further genetic tests involving larger cohorts and gene expression analysis on patients' specimens should be performed to confirm that the A allele is able to induce a significant down-regulation of *PHOX2B* expression.

Moreover, as cardiac channelopathies, mainly represented by long QT syndrome (35), are cardiac defects predisposing to sudden death, in further investigations, the role of KCNQ1, KCNH2, and SCN5A genes should be excluded.

Given our homogeneous set of patients, the present genetic data could be valid only in the Italian population. Despite the fact that the role of ethnicity in the variable incidence of SIDS among countries could be due to different socioeconomic environments and caregiving and child-rearing practices, still the virtual absence of the c.552T-c^*^161A haplotype in the Asian population, where SIDS incidence is very low, and the highest frequency in Africans, characterized by a high SIDS incidence (36, 37), sustain a role of PHOX2B in the etiology of these events. Consistently, the results reported here support the hypothesis of a wider, loss-of-function effect of *PHOX2B* common variants in the predisposition to infant life-threatening and sudden death events.

## Data Availability Statement

The datasets presented in this study can be found in online repositories. The name of the repository and accession numbers can be found below: Leiden Open Variation Database (LOVD), https://databases.lovd.nl/shared/screenings/PHOX2B, Screening IDs: 0000326133, 0000326132, 0000326136, 0000326137, and 0000326134.

## Ethics Statement

Ethical review and approval was not required for the study on human participants in accordance with the local legislation and institutional requirements. Written informed consent to participate in this study was provided by the participants' legal guardian/next of kin.

## Author Contributions

TB designed and performed experiments and wrote the manuscript. SB performed experiments. RP and AP performed clinical analysis of patients. IC data analysis and manuscript editing. All authors contributed to the article and approved the submitted version.

## Conflict of Interest

The authors declare that the research was conducted in the absence of any commercial or financial relationships that could be construed as a potential conflict of interest.
