# Supplementary material for: A Common 3′UTR Variant of the PHOX2B Gene Is Associated With Infant Life-Threatening and Sudden Death Events in the Italian Population
Source: Front Neurol. 2021 Mar 19;12:642735. doi: 10.3389/fneur.2021.642735 (PMC8017182; doi:10.3389/fneur.2021.642735)
Supplement: Supplementary file 4 [file Presentation_1.PPT]

## Slide 1
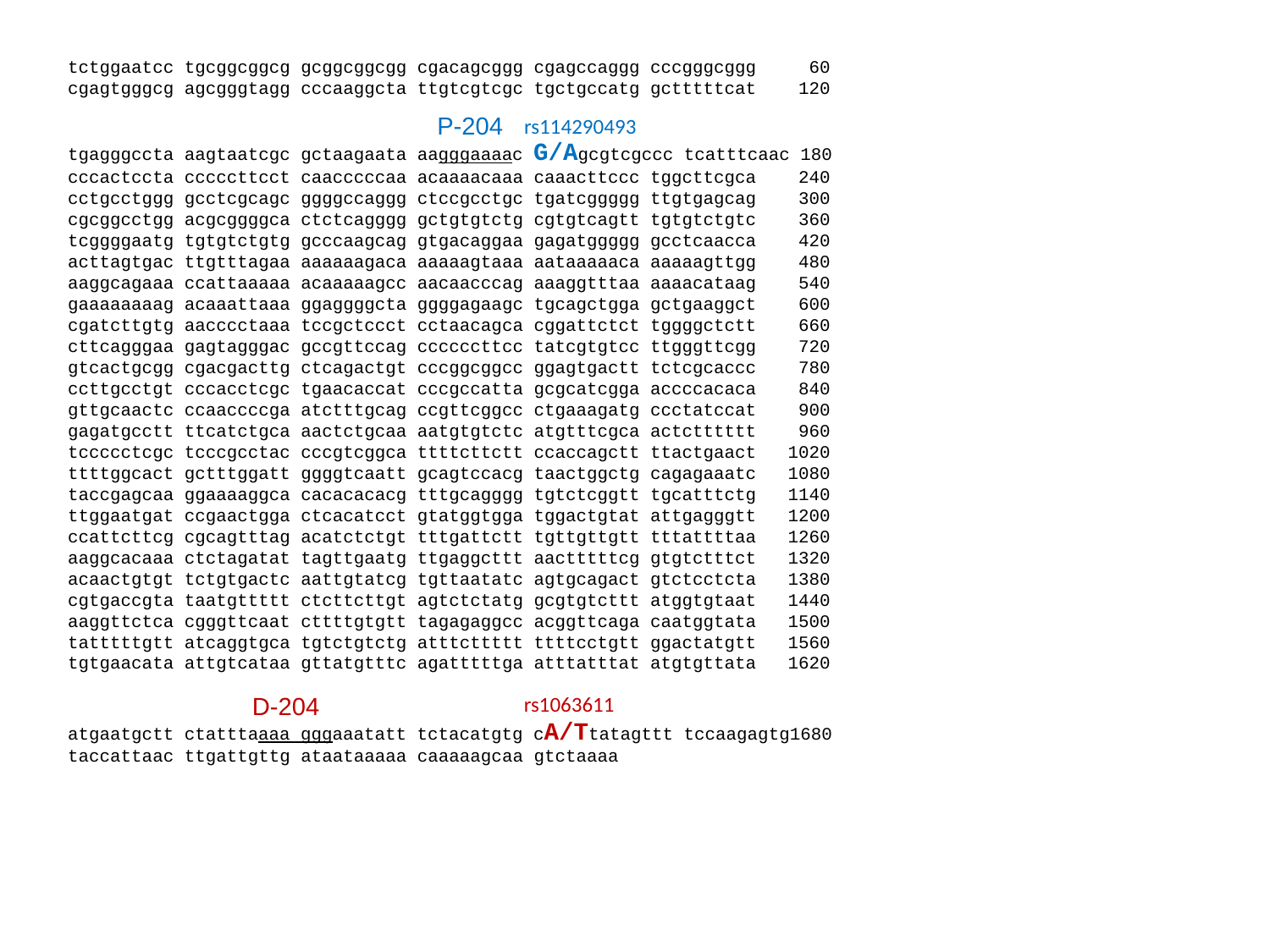

tctggaatcc tgcggcggcg gcggcggcgg cgacagcggg cgagccaggg cccgggcggg 60
cgagtgggcg agcgggtagg cccaaggcta ttgtcgtcgc tgctgccatg gctttttcat 120
tgagggccta aagtaatcgc gctaagaata aagggaaaac G/Agcgtcgccc tcatttcaac 180
cccactccta cccccttcct caacccccaa acaaaacaaa caaacttccc tggcttcgca 240
cctgcctggg gcctcgcagc ggggccaggg ctccgcctgc tgatcggggg ttgtgagcag 300
cgcggcctgg acgcggggca ctctcagggg gctgtgtctg cgtgtcagtt tgtgtctgtc 360
tcggggaatg tgtgtctgtg gcccaagcag gtgacaggaa gagatggggg gcctcaacca 420
acttagtgac ttgtttagaa aaaaaagaca aaaaagtaaa aataaaaaca aaaaagttgg 480
aaggcagaaa ccattaaaaa acaaaaagcc aacaacccag aaaggtttaa aaaacataag 540
gaaaaaaaag acaaattaaa ggaggggcta ggggagaagc tgcagctgga gctgaaggct 600
cgatcttgtg aacccctaaa tccgctccct cctaacagca cggattctct tggggctctt 660
cttcagggaa gagtagggac gccgttccag ccccccttcc tatcgtgtcc ttgggttcgg 720
gtcactgcgg cgacgacttg ctcagactgt cccggcggcc ggagtgactt tctcgcaccc 780
ccttgcctgt cccacctcgc tgaacaccat cccgccatta gcgcatcgga accccacaca 840
gttgcaactc ccaaccccga atctttgcag ccgttcggcc ctgaaagatg ccctatccat 900
gagatgcctt ttcatctgca aactctgcaa aatgtgtctc atgtttcgca actctttttt 960
tccccctcgc tcccgcctac cccgtcggca ttttcttctt ccaccagctt ttactgaact 1020
ttttggcact gctttggatt ggggtcaatt gcagtccacg taactggctg cagagaaatc 1080
taccgagcaa ggaaaaggca cacacacacg tttgcagggg tgtctcggtt tgcatttctg 1140
ttggaatgat ccgaactgga ctcacatcct gtatggtgga tggactgtat attgagggtt 1200
ccattcttcg cgcagtttag acatctctgt tttgattctt tgttgttgtt tttattttaa 1260
aaggcacaaa ctctagatat tagttgaatg ttgaggcttt aactttttcg gtgtctttct 1320
acaactgtgt tctgtgactc aattgtatcg tgttaatatc agtgcagact gtctcctcta 1380
cgtgaccgta taatgttttt ctcttcttgt agtctctatg gcgtgtcttt atggtgtaat 1440
aaggttctca cgggttcaat cttttgtgtt tagagaggcc acggttcaga caatggtata 1500
tatttttgtt atcaggtgca tgtctgtctg atttcttttt ttttcctgtt ggactatgtt 1560
tgtgaacata attgtcataa gttatgtttc agatttttga atttatttat atgtgttata 1620
atgaatgctt ctatttaaaa gggaaatatt tctacatgtg cA/Ttatagttt tccaagagtg1680
taccattaac ttgattgttg ataataaaaa caaaaagcaa gtctaaaa
P-204
rs114290493
D-204
rs1063611
