# Supplementary material for: A Common 3′UTR Variant of the PHOX2B Gene Is Associated With Infant Life-Threatening and Sudden Death Events in the Italian Population
Source: Front Neurol. 2021 Mar 19;12:642735. doi: 10.3389/fneur.2021.642735 (PMC8017182; doi:10.3389/fneur.2021.642735)
Supplement: Supplementary file 5 [file Presentation_2.PPTX]

## Slide 1
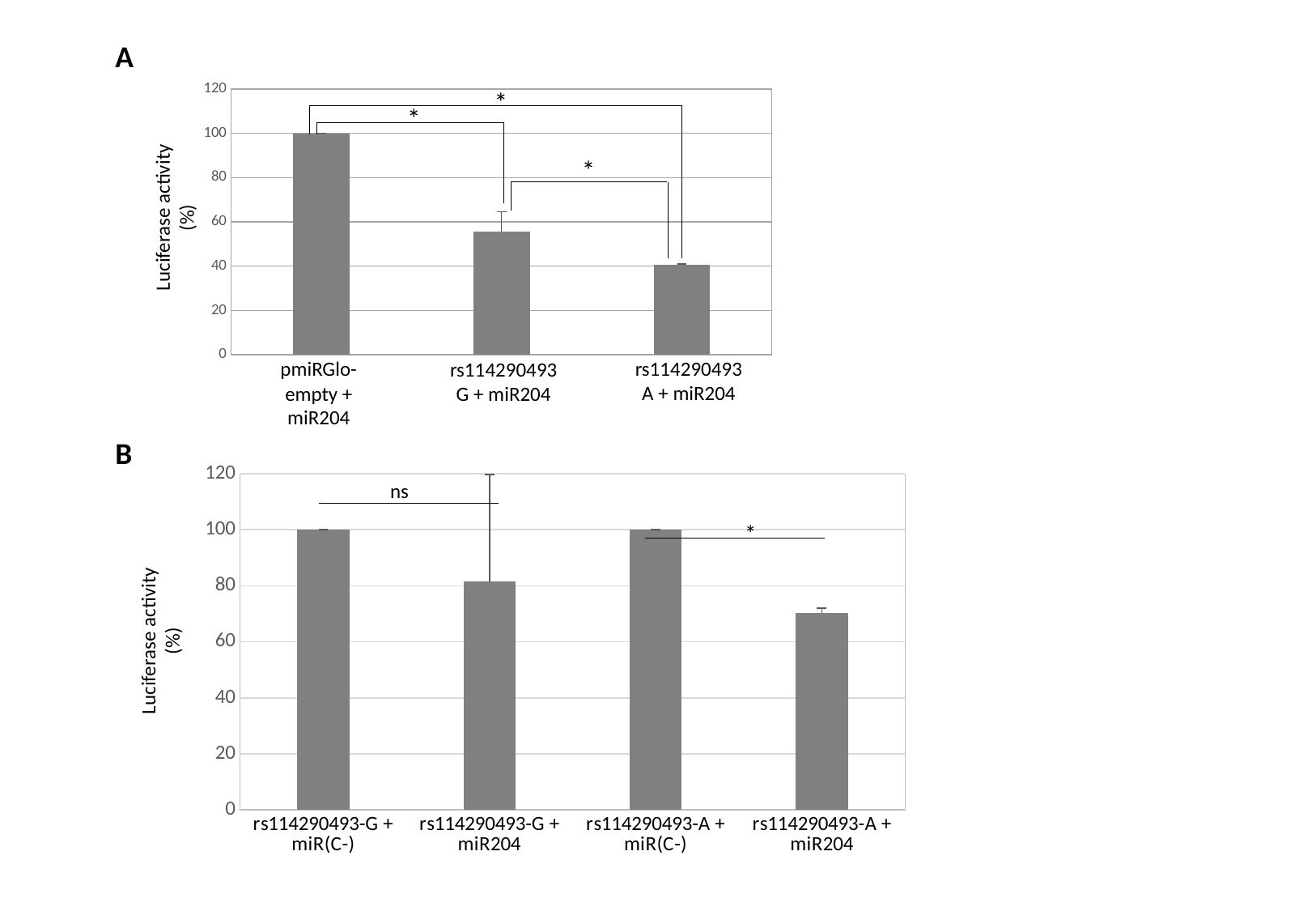

A
*
### Chart
| Category | |
|---|---|
| pmiRGloempty + miR204 | 100.0 |
| rs114290493-G + miR204 | 55.75 |
| rs114290493-A + miR204 | 40.599999999999994 |*
*
Luciferase activity (%)
rs114290493
A + miR204
pmiRGlo-empty + miR204
rs114290493
G + miR204
B
### Chart
| Category | |
|---|---|
| rs114290493-G + miR(C-) | 100.0 |
| rs114290493-G + miR204 | 81.56666666666666 |
| rs114290493-A + miR(C-) | 100.0 |
| rs114290493-A + miR204 | 70.39999999999999 |ns
*
Luciferase activity (%)
