# Supplementary material for: A Common 3′UTR Variant of the PHOX2B Gene Is Associated With Infant Life-Threatening and Sudden Death Events in the Italian Population
Source: Front Neurol. 2021 Mar 19;12:642735. doi: 10.3389/fneur.2021.642735 (PMC8017182; doi:10.3389/fneur.2021.642735)
Supplement: Supplementary file 6 [file Data_Sheet_1.docx]

**LEGENDS TO SUPPLEMENTAL FIGURES**

**Suppl. Figure 1 – Position of the SNPs lying next to miR-204 sites on the 3’UTR *PHOX2B***

Sequence of the *PHOX2B* 3’UTR showing the position of the proximal (P-204) and distal (D-204) miR-204 binding sites; in addition, the position of the two SNPs rs114290493 and rs1063611, lying nearby the P-204 and D-204 elements, respectively, is shown.

**Suppl. Figure 2 –Effects of miR-204 on c*361 alleles**

1. The effect of the two G and A alleles is comparedwith the effect of the empty vector, in the presence of miR-204. Values are the mean of three independent experiments performed in triplicate ±SD Asterisk * indicates statistical significance (Student t’test, p<0,05)
2. The graph bar shows the Luciferase activity, induced by addition of miR-204 to each construct, calculated as percentage of the value obtained following transfection of the miR-204 and compared with the negative control referred as 100. Values are the mean of three independent experiments performed in triplicate ±SD. Asterisk * indicates statistical significance (Student t’test, p<0,05)
